# Assessment of *Bacillus subtilis* Plasmid pLS20 Conjugation in the Absence of Quorum Sensing Repression

**DOI:** 10.3390/microorganisms9091931

**Published:** 2021-09-10

**Authors:** Kotaro Mori, Valeria Verrone, Ryotaro Amatsu, Kaho Fukui, Wilfried J. J. Meijer, Shu Ishikawa, Anil Wipat, Ken-ichi Yoshida

**Affiliations:** 1 Department of Science, Technology and Innovation, Kobe University, 1-1 Rokkodai, Nada, Kobe 657-8501, Japan; gtmrktr@outlook.jp (K.M.); amaryo205@gmail.com (R.A.); kahott-plie@outlook.jp (K.F.); shu@people.kobe-u.ac.jp (S.I.); 2 School of Computing, Newcastle University, 1 Science Square, Science Central, Newcastle upon Tyne NE4 5TG, UK; V.Verrone2@newcastle.ac.uk (V.V.); anil.wipat@newcastle.ac.uk (A.W.); 3 Centro de Biología Molecular ‘Severo Ochoa’ (CSIC-UAM), Universidad Autónoma, Canto Blanco, 28049 Madrid, Spain; wmeijer@cbm.csic.es

**Keywords:** *Bacillus subtilis*, cell aggregation, conjugation, fluorescence-activated cell sorting, plasmid

## Abstract

*Bacillus subtilis* conjugative plasmid pLS20 uses a quorum-sensing mechanism to control expression levels of its conjugation genes, involving the repressor Rco_pLS20_, the anti-repressor Rap_pLS20_, and the signaling peptide Phr*_pLS20_. In previous studies, artificial overexpression of *rap_pLS20_* in the donor cells was shown to enhance conjugation efficiency. However, we found that the overexpression of *rap_pLS20_* led to various phenotypic traits, including cell aggregation and death, which might have affected the correct determination of the conjugation efficiency when determined by colony formation assay. In the current study, conjugation efficiencies were determined under different conditions using a two-color fluorescence-activated flow cytometry method and measuring a single-round of pLS20-mediated transfer of a mobilizable plasmid. Under standard conditions, the conjugation efficiency obtained by fluorescence-activated flow cytometry was 23-fold higher than that obtained by colony formation. Furthermore, the efficiency difference increased to 45-fold when *rap_pLS20_* was overexpressed.

## 1. Introduction

Bacteria can adapt to various environments by acquiring new genetic information from the environment. Horizontally transferred genetic elements are propagated among a wide range of species and contribute to the plasticity of bacterial genomes. There are at least three types of horizontal gene transfer in bacteria, namely transformation, transfection, and conjugation. Conjugation is the horizontal transfer of genetic elements through the direct connection between two cells [1]. Conjugation frequently occurs across species in the natural environment, provoking the problem of spreading antibiotic-resistance genes [2].

Conjugation requires direct contact between two cells, as the DNA element is transferred from a donor cell to a recipient cell through a channel that connects them. Conjugative elements are often located on plasmids but can also be incorporated in chromosomes, known as integrative conjugative elements (ICEs). Conjugation occurs both in Gram-positive and Gram-negative bacteria. Although some differences exist, the conjugation process is composed of four conserved stages [3,4,5,6,7,8,9]. In the first stage, a donor cell attaches to a suitable recipient cell in a process named mating pair formation. This process involves proteins located on the cell surface, including adhesion proteins, which enable the contact between the donor and the recipient cells. In Gram-negative bacteria, the adhesion proteins are located at the tip of a tuberous structure called sex pili. Conjugative elements in Gram-positive bacteria apparently do not encode sex pili, instead, they encode adhesins that play an important role in the mating process. In addition, the selection of a suitable recipient cell also involves proteins playing a role in a process called exclusion that inhibits the transfer of the conjugative element between two donor cells. In the second stage, a specific form of DNA is produced in the donor cell, which is usually a single-stranded DNA (ssDNA) molecule that is transferred into the recipient cell. The generation of the ssDNA starts with the formation of a nucleoprotein complex, named the relaxosome at a specific region of the conjugative element called the origin of transfer (*oriT*). The relaxosome often contains auxiliary proteins, which assist the crucial relaxosome protein, a relaxase in carrying out a site and strand-specific nick within the *oriT* sequence. After nicking, the relaxase remains covalently attached to the 5’-site of the nicked DNA and pilots the ssDNA into the recipient cell. In the third stage, a sophisticated membrane-embedded translocation apparatus is synthesized in the donor cell, which is a type-IV secretion system that is composed of at least twelve and often more different protein subunits [9,10,11]. The relaxosome is recruited to the translocation apparatus by the interaction between the relaxase and an ATPase coupling protein on the cytoplasmic entry side of the translocation apparatus. The relaxase exerts another function by piloting the ssDNA into the recipient cell. Finally, in the fourth stage, the transferred ssDNA has to be converted into double-stranded DNA (dsDNA), for which primases are supplied by the conjugative system or the host. The genes required for conjugation are tightly regulated for at least two reasons. Firstly, the synthesis of many of the conjugation proteins imposes an energetic burden on the cell. Secondly, once the conjugation mechanism is activated, various changes occur not only to the host but also in the plasmid. Conjugative plasmids use a theta-type mechanism of DNA replication. However, during conjugation, replication switches to a rolling circle mechanism of replication to generate the ssDNA molecule that is transferred into the recipient cell [5,12]. In addition, during conjugation, the plasmids are recruited to the entry site of the translocation apparatus [13,14,15]. Furthermore, the conjugation process alters features of the surface structure as many components in the cell membrane and wall are changed drastically.

*Bacillus subtilis* represents Gram-positive bacteria as one of the best-studied species [16,17]. However, relatively little is known about its conjugative plasmids, and pLS20 is one of a few examples. pLS20 was identified in strain IFO3335 of *B. subtilis* var. natto [18], and it exerts efficient conjugation in liquid as well as on solid media [19,20,21]. Studies on regulatory mechanisms of conjugation genes of pLS20 have revealed that they are under the control of quorum sensing, involving the repressor Rco_pLS20_, the anti-repressor Rap_pLS20_, and the signaling peptide Phr*_pLS20_ [22]. The interaction of the three elements is integrated to regulate the activity of the major promoter Pc, which is located upstream of the long operon required for conjugation and for the expression of the operon. For proper expression of the operon, the promoter is controlled as follows. First, the promoter is repressed by default by Rco_pLS20_, while the intercellular quorum signaling system senses conditions favorable for conjugation. Second, different layers of regulation ensure tight repression of the promoter while allowing its rapid activation when needed [23] (for review [24]). Artificial overproduction of the anti-repressor Rap_pLS20_ is expected to override the inhibitory effects of signaling peptide Phr*_pLS20_ on the expression of the conjugation genes. Indeed, recent studies demonstrated that the ectopic expression of *rap_pLS20_* encoding anti-repressor leads to overexpression of the conjugation genes and increases moderately the levels of conjugation efficiency [24]. However, as mentioned above, artificial overexpression of conjugation genes may have adverse effects on the cell. So far, the likely negative effects of overexpression of the conjugation genes have not been assessed.

The standard method for measuring conjugation efficiency is as follows. The conjugative element and the recipient strain are labeled with different antibiotic resistance markers. The donors carrying the conjugative element and the recipients are mixed for conjugation. After a mating period, the conjugation mixture is spread on selective agar plates for donors, recipients, and transconjugants. The colony counts are used to calculate the conjugation efficiency as the ratio of transconjugants per recipient or donor. However, recently an alternative methodology has been described in which the conjugation efficiency is determined by labeling the conjugative element and the recipient cells with genes encoding different fluorescent proteins and analyzing the cells with fluorescence-activated flow cytometry and microscopy [25,26,27,28]. The methodology may also allow observation of the dynamics in cell–cell interaction [29] and the quantitative data for simulating the conjugation process in silico [30]. pLS20 conjugates efficiently in liquid media (see above) and therefore is an ideal system to determine conjugation efficiency by fluorescence-activated flow cytometry. In this study, a mobilizable plasmid and the recipient cell labeled with a different gene for fluorescent protein were subjected to fluorescence-activated flow cytometry to investigate pLS20-mediated conjugation. We re-evaluate the efficiency of pLS20 conjugation upon the enhanced production of Rap_pLS20_, and visualize possible effects of the artificial overexpression of the conjugation genes on cell aggregation.

## 2. Materials and Methods

### 2.1. Bacterial Strains, Plasmids, and Primers

The strains and plasmids used in this experiment are shown in Table 1. The primers used are listed in Table 2. The strains were cultured in LB medium (Becton, Dickinson and Company, Franklin Lakes, NJ, USA). Antibiotics were added as needed; 5 mg/L chloramphenicol, 1 mg/L erythromycin, 7.5 mg/L kanamycin, 100 mg/L spectinomycin, and 12.5 mg/L tetracycline.

### 2.2. Plasmid and Strain Construction

A recipient *B. subtilis* strain KV7 was constructed as follows. To inactivate *comK* that encodes a transcription factor essential for natural competence [36], and simultaneously introduce the tetracycline resistance cassette, DNA fragments corresponding to *comK* upstream and downstream regions were amplified by PCR using the primer pairs comK_up_F/comK_up_R and comK_Down_F/comK_Down_R (Table 2), respectively, using *B. subtilis* 168 chromosomal DNA as a template. Whereas a fragment of the *tet* gene for tetracycline resistance was amplified from plasmid pOGW [35] using a primer pair tetR_F/tetR_R (Table 2). The primers used contained 30 nucleotides overhangs to allow recombinant PCR to ligate the three fragments to place the *tet* fragment in the middle using the nested primers comK_nested_F/comK_nested_R (Table 2). The recombinant PCR product was used to transform *B. subtilis* 168 to be tetracycline resistant. The resulting strain was designated as KV7.

A donor *B. subtilis* strain KV9 was made from YNB102 by introducing the mobilizable plasmid pGR16B [32].

Another recipient *B. subtilis* strain KV10 was constructed as follows. A DNA fragment for constitutive expression of *mKATE2*, a bright red fluorescent protein, was amplified by PCR from pDG_SG51 [33] using the primer pair mKate2_F/Kate2_Kan_R (Table 2). Another PCR fragment encoding the kanamycin resistance gene was made from pANPCK [34] by PCR using the primer pair Kan_F/Kan_terminator_R (Table 2). Two more DNA fragments corresponding to *aprE* upstream and downstream regions were amplified by PCR using the primer pairs aprE_U_F/aprE_U_PrpsO_R and aprE_D_F/aprE_D_R (Table 2), respectively, from *B. subtilis* 168 chromosomal DNA. Moreover, the *aprE* upstream, the *mKATE2*, the kanamycin resistance, and the *aprE* downstream fragments were ligated in this order by recombinant PCR using the primer pair aprEU_F3_nested/aprE_D_R2_nested (Table 2). The recombinant PCR product was used to transform strain KV7 to be kanamycin resistant. The resulting strain was designated as KV10 labeled with mKATE2.

A variant of pGR16B, pGR16B_sfGFP, was constructed as follows. The DNA of pGR16B was linearized by digestion with EcoRI (Takara Bio, Shiga, Japan) and simultaneously treated with shrimp alkaline phosphatase (Takara Bio) to remove the phosphate at the 5’ end. An artificially designed genetic circuit that includes the gene for a super folder green fluorescent protein, *sfGFP* [37], was synthesized by IDT (Integrated DNA Technologies, Coralville, IA, USA) and composed to place the *sfGFP* codon-optimized for *B. subtilis* under the control of the P*veg* promoter [38] and the *amyS* terminator [39]. NEBuilder HiFi DNA Assembly (New England Biolabs, Ipswich, MA, USA) was used to insert the DNA fragment of *sfGFP* circuit into the EcoRI-linearized plasmid DNA. The resulting plasmid was introduced into *Escherichia coli* C600 to form bright green colonies on ampicillin plates, which carrying the plasmid pGR16B_sfGFP.

Another donor strain KV11 was made from YNB102 [31] by introducing the mobilizable plasmid pGR16B_sfGFP.

### 2.3. Conjugation

Conjugation was performed as described previously [31] with some minor modifications. Bacterial strains were grown overnight at 30 °C in LB agar plates containing appropriate antibiotics. One of the freshly formed colonies was inoculated into a liquid LB medium and allowed to grow at 37 °C with shaking at 200 rpm. At 2 h after the inoculation, only for the cultures of the donor strains, 1 mM IPTG was added to induce the overexpression of *rap_pLS20_*. When the optical density for the cells at 600 nm (OD_600_) reached 0.5, the donor and recipient cells were mixed in test tubes in various ratios to make a total volume of 0.5 mL. The tubes were incubated at 37 °C for 15 min without shaking to promote conjugation, and then the cells were allowed to grow again at 37 °C for 1 h with shaking at 200 rpm. The cell mixtures were diluted and spread on the appropriate agar plates to form colonies overnight at 37 °C or subjected to flow cytometric analysis as follows.

### 2.4. Flow Cytometric Analysis

**Figure A1:**
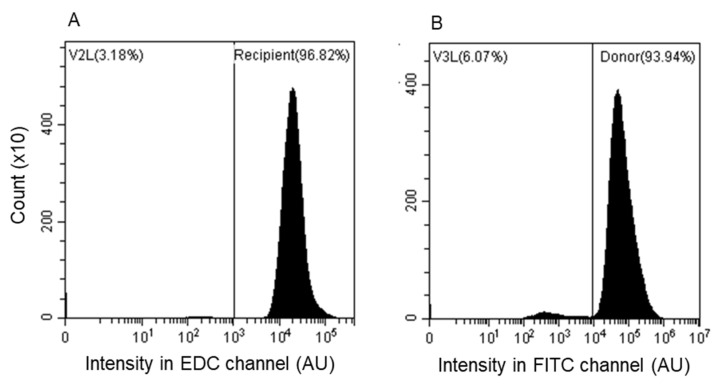
Determination of gate hierarchy. The negative populations V2L (**A**) and V3L (**B**) are defined as noise. The experiments were repeated three times with similar results and one set of representative plots is shown.

The cells of strains KV10 and KV11 after the conjugation were fixed with 4% (*v/v*) formaldehyde at room temperature for 30 min, washed once in a buffer [10 mM Tris-HCl (pH 8.0), 1 mM EDTA, 200 mM KCl, and 5% (*v/v*) glycerol], and suspended in PBS (1 mM KH_2_PO_4_, 3 mM Na_2_HPO_4_, and 155 mM NaCl). This cell fixation would not allow the biological assessment of effects of sonication but the analysis with fluorescence-activated flow cytometry. The cell suspension was subjected to brief sonication using Bioruptor UCD-250 (Cosmo Bio, Tokyo, Japan) in optimized conditions to disperse the cell aggregate into single cells as described previously [40]. Then, the cells were diluted with PBS appropriately and analyzed by the fluorescence-activated flow cytometry using CytoFlexS (Beckman Coulter, Pasadena, CA, USA), and data analysis was done using the bundled software CytoExpert. *sfGFP* was detected using an FITC channel (525/40 BP) with an excitation beam at 488 nm. *mKATE2* was detected using an ECD channel (610/20 BP) with an excitation beam at 561 nm. The flow rate was set to 0.04 mL/min to ensure that 100,000 events were detected at the rate of less than 1500 events/s. 3.18% of the KV10 events gave the ECD signal intensity below 10^3^ arbitrary units (AU) and thus had to be excluded as “dark events (V2L)” from further analysis (Figure A1A), while the remaining 96.82% were saved, which allowed enough number of events to perform the analysis. In the case of KV11 (Figure A1B), similarly, 6.07% gave the FITC signal intensity below 10^4^ AU to be also excluded as the other dark events (V3L), and the remaining 93.34% was saved. The saved events were determined to be the significant objects of analysis and designated as “bright events”.

### 2.5. Microscopy

Strains KV7 and KV9 were grown to OD_600_ = 0.5, mixed for mating when needed, and subjected to LIVE/DEAD staining, which was performed using LIVE/DEAD^®^ BacLight Bacterial Viability Kit (Thermo Fisher Scientific, Waltham, MA, USA) according to the procedure as instructed in the user’s manual. After staining, an aliquot of the cell suspension was dropped onto a slide glass coated with 1% agar, covered with a cover glass, and examined under a fluorescence microscope Eclipse Ti (Nikon, Tokyo, Japan). The red fluorescence was observed using the FM4-64 channel with an analog gain of 17.1×, exposure time of 1 s, and ND filter of 4, whereas the green fluorescence was observed using the GFP channel with an analog gain of 17.1×, exposure time of 1 s, and ND filter of 16. The micrographs were analyzed using Image-J (https://imagej.nih.gov/ij/index.html, last accessed on 9 September 2021).

## 3. Results

### 3.1. Conventional Colony Formation Assay

The conjugation efficiency of transferring pLS20 itself may be misestimated because a generated transconjugant can serve as a donor that transfers the plasmid in the second round of conjugation. The following strategy was employed to avoid this.

Besides being able to transfer itself, pLS20 can mobilize several small rolling circle plasmids [19,20]. Contrary to a conjugative plasmid, a mobilizable plasmid is unable to undergo a second round of transfer since it requires the co-residence of a conjugative plasmid. For our experimental system, therefore, we used a donor strain, KV9, containing pLS20catΔoriT and pGR16B (labeled with *cat* and *erm*, respectively). Plasmid pLS20catΔoriT is a derivative of pLS20cat lacking *oriT* and therefore is defective in conjugative self-transfer. However, it can mobilize the co-resident small rolling circle plasmid pGR16B that contains the *oriT* of pLS20 [31,32].

Besides harboring pLS20ΔoriT and pGR16B, the donor strain KV9 contains a copy of the *rap_pLS20_* gene on its chromosome under the control of an IPTG-inducible promoter, which allows conditional overexpression of the conjugation genes [22]. The tetracycline-resistant and competence deficient strain KV7 (Δ*comK*::*tet*) was used as the recipient strain. After mating, which was performed using different recipient:donor ratios, the cell suspensions were diluted appropriately and plated on agar media supplemented with different antibiotics to select for donors (*cat* and *erm*), recipients (*tet*), and transconjugants (*erm* and *tet*). After overnight growth, the colony-forming units (CFU) were calculated from the number of colonies on the plate. Since the CFU values depend on the cell concentration, the values affected by subtle differences in cell concentration, which are inevitable for each experiment, were normalized by OD_600_ of each mating mixture (CFU/OD_600_).

**Figure 1:**
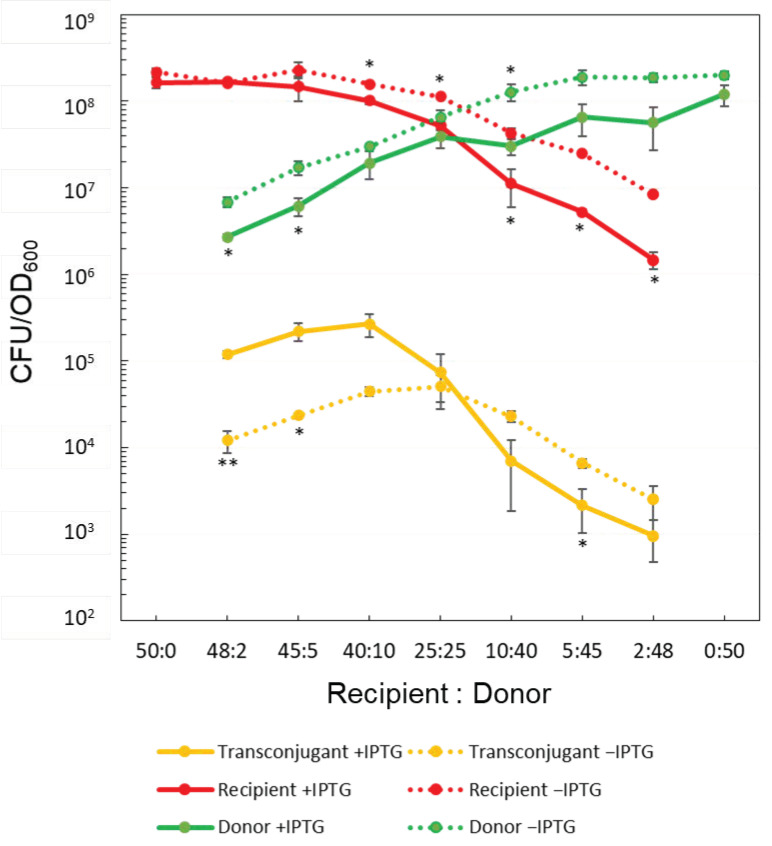
Summary of colony formation assays. Strains KV7 and KV9 were grown to OD_600_ = 0.5 (+/− IPTG only for KV9), mixed in various ratios for conjugation, and spread on agar plates to form colonies to calculate the respective CFU/OD_600_ under the appropriate selective conditions; both *cat* and *erm* for donors, *tet* for recipients, and both *erm* and *tet* for transconjugants. The values are the means ± SD of three independent experiments with similar results. Statistical significances by *t*-test due to the presence of IPTG are indicated (* *p* < 0.05; ** *p* < 0.01).

As shown in Figure 1, the CFU/OD_600_ of donors, recipients, and transconjugants in each ratio increased or decreased differently with and without IPTG addition for donors. Three noteworthy changes were observed as follows:

(i) The CFU/OD_600_ values of both donors and recipients tended to decrease when the donors had been grown in the presence of IPTG compared to its absence.
(ii) In the presence of IPTG, the CFU/OD_600_ of transconjugants was higher only when the concentration of recipients exceeded that of donors, whereas, in the absence of IPTG, the CFU/OD_600_ of transconjugants gave an apparent peak at recipients: donors = 25:25, and the value similar to that were found in the presence of IPTG.
(iii) As the number of donors exceeded that of recipients, the CFU/OD_600_ of transconjugants showed a tendency to be lower in the presence of IPTG than in its absence.

Mating pair formation between donor and recipient cells may result in aggregated cells from which colonies develop on the plate. In this work, we refer to the aggregated cells as cell aggregates. The formation of aggregated cells may explain the apparent reduction in the number of colonies. The adhesion proteins within the conjugation apparatus could facilitate cell aggregation, as observed in many other bacterial species [41]. The tuberous structure so-called sex pili have not yet been identified in the pLS20 system; however, a gene functioning for adhesion was identified within the conjugative element recently [42], and hence overexpression of the conjugation genes may lead to more frequent cell–cell aggregation. Thus, we created a value named Cell Aggregate Rate (CAR), which is expressed as Equation (1), where the “expected CFU” is the CFU of donors and recipients calculated according to the respective mixing ratios. The CAR value represents the average number of cells in a possible cell aggregate. The “actual CFU” is the CFU of donors and recipients that appeared on the plates containing respective antibiotics after the conjugation.

CAR = (expected CFU/OD_600_)/(actual CFU/OD_600_) (1)

In the absence of cell aggregation, each cell forms a single colony. In this case, the CAR value will be 1. However, if there is cell aggregation, the actual CFUs will be lower than the expected CFUs resulting in CAR values higher than 1. Therefore, the CAR value is a measure of cell aggregation.

**Figure 2:**
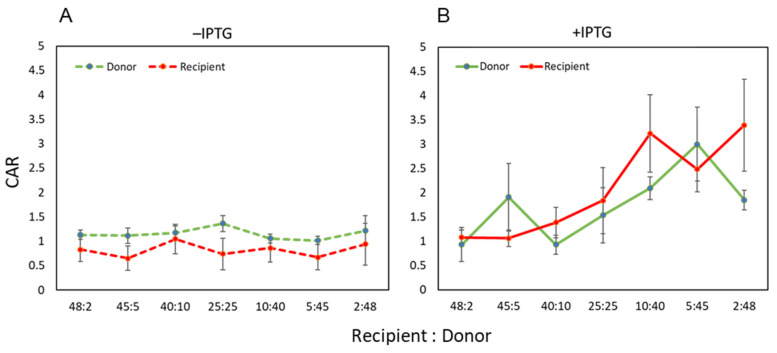
The CAR values without (**A**) and with (**B**) IPTG. The values are the means ± SD of three independent experiments.

In the absence of IPTG and without overexpression of the conjugation genes in donors, the CAR values of both donor and recipient cells calculated for various ratios of mating were consistently found to be around 1 (Figure 2A), indicating that only a minor fraction of the cells formed aggregates. On the other hand, in the presence of IPTG, the CAR values of both donor and recipient cells were higher than 1, and it augmented as the ratio of donors in the mixture increased (Figure 2B). These results suggested that overexpression of the conjugation genes indeed resulted in cell aggregates and that the ratio of donor versus recipient cells influenced the average number of cells constituting a cell aggregate.

### 3.2. Microscopic Observation

**Figure 3:**
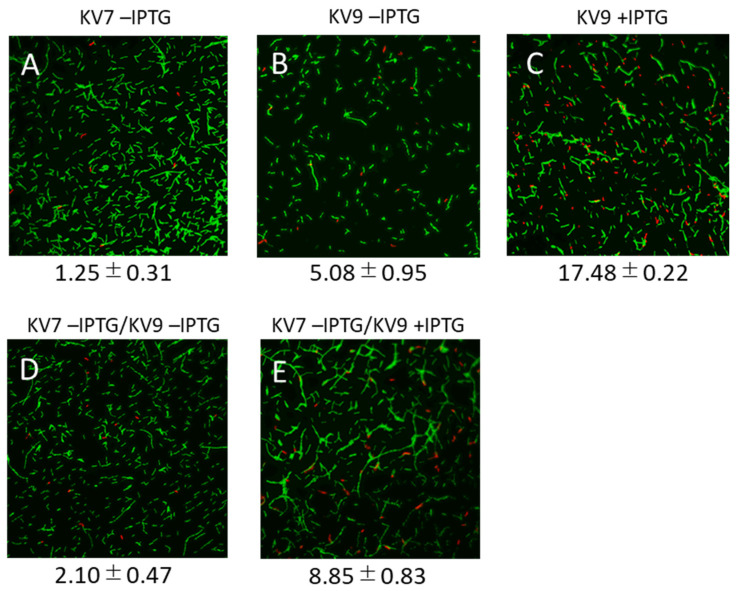
LIVE/DEAD staining of strains KV7 and KV9. KV7 (**A**) and KV9 (**B**,**C**) were grown individually to OD_600_ = 0.5 with (**C**) and without (**A**,**B**) IPTG, and then subjected to the LIVE/DEAD staining to distinguish the dead cells in red and living ones in green under 400× microscopy. The same volumes of KV7 and KV9 cultures were mixed for conjugation and stained (**D**,**E**), where KV9 was grown with (**E**) and without (**D**) IPTG. The values beneath each panel are the percentage of dead cells calculated from four independent experiments with similar results.

Besides cell aggregate formation, overexpression of the conjugation genes may cause other effects. For instance, it may pose a burden that can affect the survival of donors. To test possible negative effects of overexpression of the conjugation genes, we analyzed mating cells by microscopy in the presence of SYTO9 and propidium iodide, which labels viable and dead cells green and red, respectively (Figure 3).

Only a few of the KV7 recipient cells (1.25 ± 0.31%) were stained red with propidium iodide, showing that under these conditions, only a minor fraction of the cells were dead or had an impaired cell membrane integrity (Figure 3A). Compared to KV7, the percentage of red-stained cells of the donor KV9 was about 4-fold higher (5.08 ± 0.95%, see Figure 3B; significant difference at *p* < 0.01), indicating that the presence of pLS20ΔoriT and pGR16B in KV9 caused an increased frequency of cell death. The percentage of dead cells of KV9 increased to more than 17% when cells were grown in the presence of IPTG to overexpress the conjugation genes (Figure 3C; *p* < 0.01). In addition, the cells showed a tendency to form chains. A known phenomenon associated with conjugation is “lethal zygosis” in which donors kill recipients [43]. To test if lethal zygosis occurs under normal conditions or when conjugation genes are overexpressed, we applied the same procedure to mating cultures. The following percentages of red-stained cells would be expected in the absence of lethal zygosis for a 25:25 (donor:recipient) mating culture based on extrapolating the results obtained with monocultures (note that the KV7 and KV9 cultures become diluted 2-fold when mixed). Without IPTG, the red-stained cells would be (1.25 + 5.08)/2 = 3.17 (%), and with IPTG this number would be (1.25 + 17.48)/2 = 9.37 (%). The experimentally obtained results were similar to the extrapolated results: the percentages of red-stained cells were 2.10 ± 0.47% and 8.85 ± 0.83% in the absence (Figure 3D) and presence (Figure 3E) of IPTG, respectively. This result indicates, therefore, that overexpression of the conjugation genes did not induce the death of recipient cells by lethal zygosis. On the other hand, the microscopic observation did not clarify the formation of cell aggregates since it was difficult to distinguish between cells that were coincidentally nearby and those specifically attached. However, the containing donor cells grown in the presence of IPTG give the impression that cells tend to be connected in chains (Figure 3D,E).

### 3.3. Assessment by Fluorescence-Activated Flow Cytometry

We next performed 2-color fluorescence-activated flow cytometry experiments to analyze conjugation at the single-cell level. In these experiments, KV11 carrying both pLS20catΔoriT and a derivative of the mobilizable plasmid pGR16B, pGR16B_sfGFP labeled with *sfGFP* encoding a green fluorescent protein (Table 1), was used as donor strain, and KV10, which has a chromosomal copy of *mKATE2* encoding for a red fluorescent protein, was used as the recipient strain. Therefore, transconjugants express both *sfGFP* and *mKATE2*. The FITC and ECD channels were used to detect fluorescence of *sfGFP* and mKATE2, respectively.

**Figure 4:**
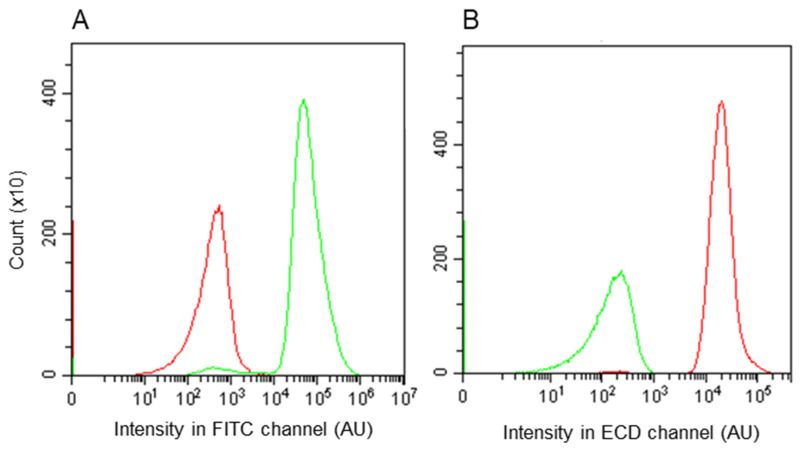
Histograms of individual KV10 (red line) and KV11 (green line) events counted for FITC (**A**) and ECD (**B**) channels. The *Y*-axis corresponds to the number of events. The *X*-axis corresponds to the intensity of fluorescence. The experiments were repeated three times with similar results, and one set of representative plots is shown.

Before performing mating between donors and recipients, individual cultures of KV11 (donor) and KV10 (recipient) were subjected to fluorescence-activated flow cytometric analysis (Figure 4) to determine the cut-off values to avoid false-positive registrations. The fluorescence signals of the vast majority of KV11 cells expressing *sfGFP* green-fluorescent protein gave intensities higher than 10^4^ AU in the FITC channel, and all the KV10 cells expressing the *mKATE2* red-fluorescent protein displayed fluorescence levels lower than 10^4^ AU. In the case of the ECD channel, all *mKATE2* and *sfGFP* expressing cells gave intensities higher and lower than 10^3^ AU, respectively. Hence, the cut-off intensities were set to 10^4^ and 10^3^ AU for the FITC and ECD channels, respectively. The cells detected with fluorescence levels higher and lower than the cut-off intensities are referred to as bright and dark events, respectively.

The procedure to determine conjugation efficiencies based on fluorescence-activated flow cytometry is given in the Materials and Methods. The conjugation was performed using KV11 grown in the presence and absence of 1 mM IPTG. In addition, the conjugation cultures were treated with/out brief sonication in the conditions optimized to separate aggregated cells into single cells [40]. When the bright events are plotted in a graph using the green fluorescence of *sfGFP* intensity on *X*-axis (FITC channel) and the red fluorescence of *mKATE2* intensity on *Y*-axis (ECD channel), the donor cells and recipient cells would correspond to events falling in the lower right (LR, bright green) and upper left (UL, bright red) areas, respectively. The events falling in the upper right (UR) area that are both bright green and red would correspond to either transconjugants or to aggregated cells containing both donor and recipient cells.

**Figure 5:**
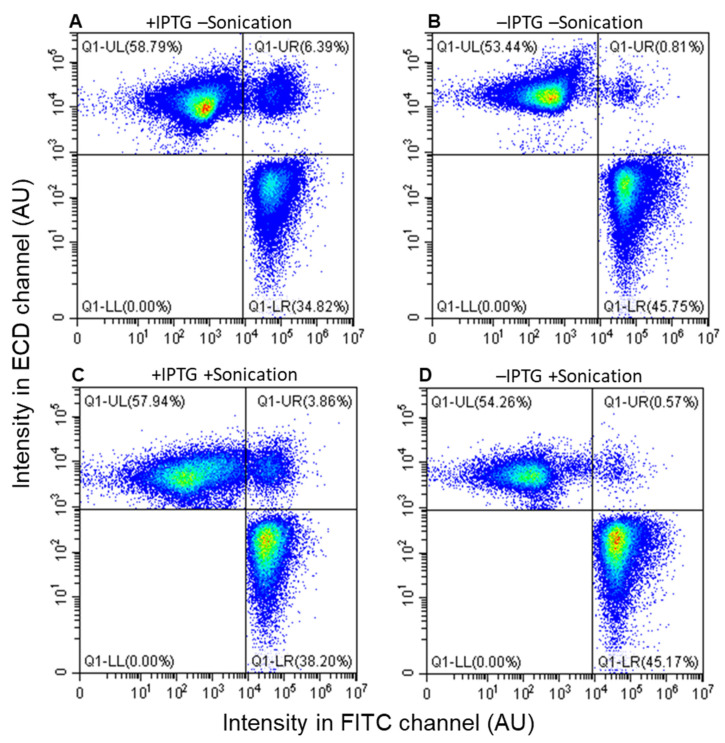
Two color plots after the mating between KV10 and KV11 cells. Mating was performed with (**A**,**C**) or without (**B**,**D**) IPTG, and then treated with (**C**,**D**) or without (**A**,**B**) a brief sonication. In response to the density of events, the heat map is set up so that the sparsest areas are blue, and as they become denser, the wavelength of visible light becomes longer from green, yellow, orange, and then to red. The experiments were repeated three times with similar results, and one set of representative plots is shown.

**Figure A2:**
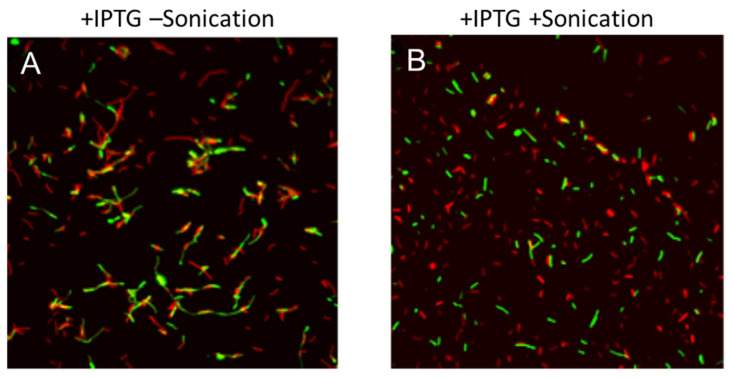
Microscopic observation of cells after mating. KV11 was grown in the presence of IPTG and mixed with KV10 for conjugation. After fixation with formaldehyde, the cells were treated without (**A**) and with (**B**) sonication and examined under a fluorescence microscope. The experiments were repeated three times and the representative results are shown, merging the images of green and red fluorescence of *sfGFP* and *mKATE2* with that of the optical microscope.

Figure 5 shows graphics of representative results of fluorescence-activated flow cytometry experiments performed in the presence (panels A and C) and absence (panels B and D) of IPTG. Previously, it has been shown that a gentle sonication treatment dispersed aggregated cells [40]. Therefore, we performed the experiment with (panels C and D) or without a sonication step (panels A and B), which would allow us to discriminate if the events falling in the UR quadrant (both bright green and red) correspond to transconjugants or to a combination of transconjugants and aggregated cells. When grown in the absence of IPTG, the sonication treatment caused a minor decrease in the percentage of events falling in the UR quadrant from 0.81 to 0.57%. In the presence of IPTG, sonication caused the percentage of dual red/green events to fall from 6.39 to 3.86%. These results indicate, therefore, that the events in the UR quadrant correspond to a mixture of transconjugant cells and aggregated cells that may be composed of donor, recipient, and perhaps also transconjugant cells. This conclusion was supported by microscopic analysis (Figure A2). Hence, the gentle sonication treatment was important to determine the percentage of transconjugants.

**Figure 6:**
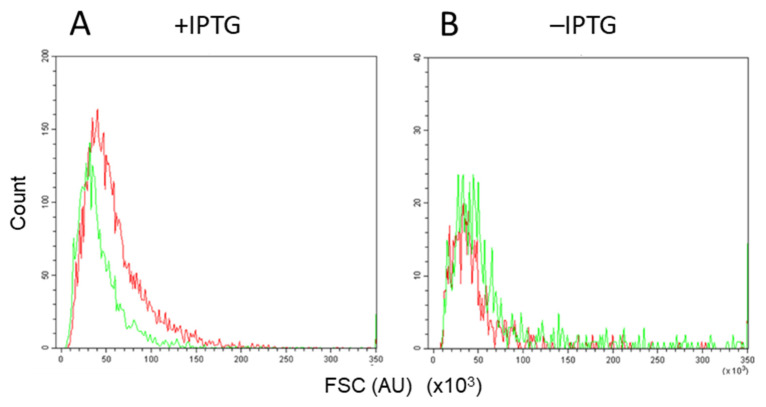
Distribution of particle size (FSC value) of events in the UR quadrants in the presence (**A**) and absence (**B**) of IPTG. The green and red lines are distributions with and without brief sonication, respectively. The experiments were repeated three times with similar results, and one set of representative plots is shown.

The FSC value (or forward scatter parameter) indicates the light reflected by a particle counted in fluorescence-activated flow cytometry, and in general, it is a measure of particle size. Contrary to cells grown in the presence of IPTG, no substantial difference was observed in the distribution of the particle sizes (FSC value) of events in the UR quadrant with and without sonic treatment for cells grown in the absence of IPTG (compare panels A and B of Figure 6). This result is consistent with the CAR value being almost equal to 1 when cells were grown in the absence of IPTG (Figure 2A), suggesting that excessive cell–cell aggregation does not occur under native mating conditions.

On the other hand, when the conjugation genes were overexpressed, several lines of evidence suggested that overexpression of the conjugation genes resulted in the formation of aggregated cells. In the presence of IPTG, the CAR value was higher than 1 (Figure 2B) in the colony assay. Sonication of samples prior to flow cytometry analysis caused the red/green dual labeled events to drop from 6.39 to 3.86% (Figure 5A,C).

Based on the populations of events in the UL and UR quadrants with sonic treatment, we deduce that the KV10 recipients acquired the mobilizable plasmid pGR16B_sfGFP with an efficiency (transconjugants/recipients) of 1.04% [0.57%/(0.57% + 54.26%) = 0.0104] when the experiment was performed under native conditions. According to this methodology, the conjugation efficiency was 6-fold higher when the conjugation genes were overexpressed (i.e., 6.25% [3.86%/(3.86% + 57.94%) = 0.0625]).

### 3.4. Accurate Assessment of Conjugation Efficiency

The artificial expression of *rap_pLS20_* overrides the quorum sensing down-regulation of the conjugation genes and causing conjugation genes to become overexpressed [24]. Based on the results of colony formation assay, the conjugation efficiencies were calculated to be 0.045% and 0.14% when donor and recipient cells were mixed at the 25:25 ratio and the donor was grown in the absence and presence of conditions to overexpress the conjugation genes, respectively (Figure 1). Thus, according to both methodologies, overexpression of the conjugation genes results in an increase in conjugation efficiency. However, these efficiencies were around 23- and 45-fold (1.04%/0.045% = 23.1 and 6.25%/0.14% = 44.6) lower than the corresponding efficiencies obtained by the fluorescence-activated flow cytometric analysis, respectively. In addition, in the case of the colony formation assay, the efficiency was elevated 3-fold (0.14%/0.045% = 3.1) higher upon the artificial overexpression of conjugation genes, which was half of the elevation of 6-fold calculated as above from the results of fluorescence-activated flow cytometric analysis.

## 4. Discussion

A conventional methodology to study conjugation efficiencies is based on a colony formation assay in which, after mating, cells are spread on plates selective for transconjugants, recipient, and donor cells. However, conjugation efficiencies obtained in this way may not be accurate for several reasons. For instance, the first step of the conjugation process involves the attachment of donor to recipient cells, which may result in cell aggregation to form cell aggregates. Since a cell aggregate composed of multiple cells will result in a single colony, the number of colonies does not reflect the number of cells preventing the accurate calculation of CFUs. This number of CFUs may provoke inaccurate determination of the conjugation efficiency, and this effect can particularly be pronounced under conditions in which the conjugation genes are overexpressed to enhance the cell–cell adhesion. In addition, under the standard colony assay, sequential transfer events of a conjugative plasmid may happen during the mating period. i.e., a plasmid transferred in an initial event from a donor to a recipient cell may be subsequently transferred from the transconjugant cell to another recipient cell. Moreover, the colony assays take two days and require many plates. Therefore, this method is relatively time-consuming, labor-intensive, and expensive.

In this study, we addressed these deficiencies to devise a system allowing the potentially more accurate determination of conjugation efficiency using a fluorescent protein-based approach combined with an experimental setup allowing only a single conjugation event during the mating period. Thus, instead of investigating the self-transfer of the conjugative plasmid pLS20cat, we analyzed the pLS20-mediated single transfer of the co-resident plasmid pGR16B. For these experiments, a self-transfer-defective derivative of pLS20cat, pLS20catΔoriT, was used. Before testing the conjugation efficiencies using the fluorescence-activated flow cytometry, we studied the possible aggregation of donor and recipient cells during mating pair formation with/out overexpressing the conjugation genes due to ectopic production of anti-repressor Rap_pLS20_. In our experimental system, *rap_pLS20_* is designed to be induced by the addition of IPTG, but previous studies have confirmed that the only genes affected by this are the conjugation genes of pLS20, and that the addition of IPTG itself does not significantly affect other cellular functions [22], strongly indicating that the effects observed here are due to the expression of *rap_pLS20_*. Comparison between the actual and the expected CFUs supported the cell aggregate formation, which was most notable when the conjugation genes were overexpressed (Figure 2). In addition, the lower recipient:donor ratio was, more cells seemed to participate in cell aggregates (Figure 3B).

Apparent aggregation of multiple cells could be observed under the microscope. However, similar cell–cell adhesion was also observed in negative control samples (Figure 2A showing recipient cells alone). It was difficult to distinguish between aggregated cells and accidental cell-to-cell adhesion. In this respect, it is worth mentioning that *B. subtilis* cells can adhere to each other in the sessile phenotype [44], which hampered the clear visualization of mating-pair formation-induced aggregation. However, microscopic analysis revealed another intriguing fact associated with the artificial overexpression of the conjugation genes. These conditions provoked increased percentages of cell death in donor cells as deduced from the LIVE/DEAD staining assays. Although some increased cell death was observed for donor cells during standard conjugation experiments, no significant increase was seen in cell death of the recipient cells within mating pair mixtures. These results indicate that cell death is not the consequence of lethal zygosis. Instead, it is more likely that it is a direct or indirect consequence of the burden posed on the donor cells due to the expression of the conjugation genes, which was enhanced obviously upon their overexpression.

The fluorescence-activated flow cytometric assay also suggested the possible formation of cell aggregate because the additional brief sonication treatment resulted in a decrease in the percentage of bright red/green double fluorescence events (Figure 5). Furthermore, this suggestion was supported by the shift in the particle size distribution caused by sonication (Figure 6A). Together, these results indicate that artificial overexpression of the conjugation genes provoked cell aggregate formation. This discovery will contribute to our understanding of the dynamics of conjugation. Due to cell aggregate formation, the conventional assay based on colony formation results in inaccurate determination of the conjugation efficiency and evaluation of the effect of overexpression of the conjugation genes.

In addition, this study also demonstrated that the colony formation-based method underestimated the efficiency of conjugation when the conjugation genes were not overexpressed artificially. The conjugation efficiency determined by the fluorescence-activated flow cytometry analysis was 23-fold higher than that of the conventional method based on colony formation. At present, we do not have a conclusive answer to explain this significant difference. However, there is at least one important difference between the fluorescence-based and colony formation-based assay. In the fluorescence-based assay, a transconjugant is defined as a cell that is doubly labeled green and red because such a cell is derived from a recipient cell that has received a copy of the mobilizable plasmid and thus expresses both the red and green fluorescent proteins. However, contrary to the transconjugants detected in the colony formation assay, it does not automatically imply that the mobilizable plasmid will establish itself in the long term in the cell that have received it.

Taken together, the results obtained here showed that the determination of the conjugation efficiencies using the colony-forming assays was not accurate. We demonstrated that conjugation efficiencies were measured efficiently, reproducibly, and rapidly using the fluorescence-activated flow cytometry-based approach. Notably, the sonication treatment was necessary to disperse aggregated cells into single cells. Therefore, the fluorescence-activated flow cytometry will be a powerful tool to produce quantitative data that enables the comparison of the effects of various conditions on conjugative transfer, which may be crucial for mathematical modeling and computer simulations. Some models for conjugation have been proposed, but most of them exclude spatial cell behavior and reproduce it based on one-to-one cell mating by fixing cells in a two-dimensional or pseudo-three-dimensional space, for example, in a biofilm [30,45]. In contrast, our present work can propose a three-dimensional model in a liquid medium, where multiple cells have the opportunity to make contact and then forming cell aggregates allowing conjugation, which may provide a new mathematical concept for modeling conjugation.

## Figures and Tables

**Table 1:** Bacterial strains and plasmids used in this study.

Strain	Description	Source or Reference
*E. coli*		
C600	Plasmid cloning host	Laboratory stock
*B. subtilis*		
168	*trpC2*	Laboratory stock
YNB102	*trpC2*, *amyE*::(P*spank*-*rap_pLS20_ spc*), pLS20catΔoriT	[31]
KV7	*trpC2*, Δ*comK*::*tet*	This study
KV9	*trpC2*, *amyE*::(P*spank*-*rap_pLS20_ spc*), pGR16B, pLS20catΔoriT	This study
KV10	*trpC2*, Δ*comK*::*tet*, *aprE*::(Pr*psO*-*mKATE2 kan*)	This study
KV11	*trpC2*, *amyE*::(P*spank*-*rap_pLS20_ spec*), pGR16B_sfGFP, pLS20catΔoriT	This study
Plasmid		
pLS20catΔoriT	pLS20cat lacking the *oriT* region	[31]
pGR16B	*erm*, *amp*	[32]
pGR16B_sfGFP	pGR16B carrying *sfGFP*	This study
pDG-SG51	*mKATE2*	[33]
pANPCK	*kan*	[34]
pOGW	*tet*	[35]

**Table 2:** Primers used in this study.

Primer	Sequence (5′-3′)
aprE_D_F	cggaaggatactacatcctggttaatcaacgtacaagcagctgcac
aprE_D_R	ggccgagcagtattcgaatgtcaag
aprEU_F3_nested	caccgagctcatagcttgtcgcgatcacctcatcc
aprE_D_R2_nested	tgctttcgctgattacaacattggtgacgctgcct
aprE_U_F	ccggtacttgccaccacatcataac
aprE_U_PrpsO_R	aatttgcgtgcgttgcaagttatttccgcactctcgctatttccgtagagactcg
comK_up_F	tgaaggattggcttattcgctctgc
comK_up_R	cagtatttcatcacttatacaacaactaataatctatcatctgtttttg
comK_Down_F	gcctggcagttccctactctcgcatgcgtgagctcggggaacggtattag
comK_Down_R	atcgaagatctgcctactgaacaaatc
comK_nested_F	gcttgagcgctgcatattctttagagagcg
comK_nested_R	gttgtaaaagcggcgcttccgtatttgccg
Kan_F	ggatagactccaccagaagagccgcaagcttacgataaacccagc
Kan_terminator_R	ccaggatgtagtatccttccgaaaaaatcccgccgctggcgggattttaactaggtactaaaacaattcatcc
mKate2_F	gggctaaatatgatttggaggtgaaacaggatgtcagaactaatcaaagagaatatg
mKate2_Kan_R	gctcttctggtggagtctatcctataaacgcagaaaggcccacccgaag
tetR_F	ttgttgtataagtgatgaaatactg
tetR_R	atgcgagagtagggaactgccaggc

## Data Availability

The data presented in this study are available on request from the corresponding author.
